# Dose-Adjusted EPOCH-R in Aggressive B-Cell Lymphomas: Efficacy, Molecular Prognostic Factors, and Real-World Outcomes from a Multicenter Turkish Cohort—A Turkish Oncology Group (TOG) Study

**DOI:** 10.3390/medicina62061117

**Published:** 2026-06-08

**Authors:** Mehmet Mutlu Kidi, Hatice Asoglu, Metehan Soysal, Tolga Koseci, Ismail Oguz Kara, Berksoy Sahin, Semra Paydas, Musa Barış Aykan, Nuri Karadurmus, Ibrahim Barista, Serkan Akin, Fatih Kus, Meltem Olga Akay, Hakan Kalyon, Can Boga, Hakan Ozdogu, Ertugrul Bayram

**Affiliations:** 1 Department of Medical Oncology, Faculty of Medicine, Cukurova University, Adana 01250, Türkiye; 2 Department of Medical Oncology, Gülhane Training and Research Hospital, Ankara 06010, Türkiye; 3 Department of Medical Oncology, Hacettepe University, Ankara 06230, Türkiye; 4 Department of Hematology, Koc University Hospital, Istanbul 34010, Türkiye; 5 Department of Hematology, Baskent University Adana Dr. Turgut Noyan Hospital, Adana 01250, Türkiye

**Keywords:** dose-adjusted EPOCH-R, diffuse large B-cell lymphoma, primary mediastinal B-cell lymphoma, double-expressor lymphoma, double-hit lymphoma, molecular prognosis, real-world evidence

## Abstract

*Background and Objectives*: Comprehensive real-world data on dose-adjusted EPOCH-R (DA-EPOCH-R) incorporating molecular prognostic stratification remain limited. We evaluated the long-term efficacy, safety, and prognostic determinants of DA-EPOCH-R in a multicenter Turkish cohort. *Materials and Methods*: This retrospective study included 140 patients with aggressive B-cell lymphoma (diffuse large B-cell lymphoma [DLBCL], *n* = 81; primary mediastinal B-cell lymphoma [PMBL], *n* = 39; other, *n* = 20) treated with DA-EPOCH-R at five academic centers (2015–2020). Molecular profiling included immunohistochemistry (MYC, BCL-2, BCL-6) and fluorescence in situ hybridization (FISH). Survival was estimated by Kaplan–Meier analysis with Cox regression for prognostic factors. *Results*: At a median follow-up of 50.1 months, 5-year overall survival (OS) and event-free survival (EFS) rates were 71.3% and 66.3%, respectively (complete response rate: 68.6%). Molecular subtypes included double-expressor (DEL; *n* = 39), triple-expressor (TEL; *n* = 21), double-hit (DHL; *n* = 17), and triple-hit lymphoma (THL; *n* = 11). Five-year OS by IPI risk group ranged from 88.6% (low) to 49.4% (high) (*p* = 0.005). DEL status did not confer inferior OS (*p* = 0.738), whereas DHL and THL had markedly poor outcomes (*p* < 0.001). In multivariate analysis, IPI ≥ 3 (HR 2.54; *p* = 0.007) and *MYC* FISH rearrangement (HR 3.62; *p* < 0.001) independently predicted inferior OS. Grade 3–4 neutropenia occurred in 57.1%, with no grade 3–4 cardiotoxicity. *Conclusions*: DA-EPOCH-R provides favorable long-term outcomes in aggressive B-cell lymphomas. DEL status did not confer a survival disadvantage, an association that is hypothesis-generating and requires confirmation, as the present design cannot establish a causal mechanism. FISH-defined DHL/THL remain associated with dismal outcomes, warranting novel therapeutic strategies.

## 1. Introduction

Large B-cell lymphomas constitute the most common subtype of non-Hodgkin lymphoma, accounting for approximately 30% of all diagnoses, with nearly 150,000 new cases reported worldwide each year [1]. The introduction of R-CHOP immunochemotherapy has established a curative standard for this disease, with long-term disease control achieved in more than 60% of patients [1,2]. This constitutes a significant therapeutic milestone, given that the majority of patients are diagnosed with advanced-stage disease [1]. Outcomes remain particularly unfavorable for patients with primary refractory disease following R-CHOP; the international SCHOLAR-1 study reported a median OS of approximately 6 months in this population, underscoring a critical unmet therapeutic need [1,3]. Advances in molecular characterization over the last two decades have uncovered substantial biologic heterogeneity within diffuse large B-cell lymphoma (DLBCL), demonstrating that this entity comprises molecularly distinct subtypes with divergent clinical trajectories [1,4].

The clinical behavior of DLBCL is fundamentally governed by its molecular and genetic landscape. Through gene expression profiling, two principal molecular subgroups have been delineated: the germinal center B-cell-like (GCB) and activated B-cell-like (ABC) subtypes. Patients with ABC-subtype disease face a considerably worse prognosis, with reported 3-year progression-free survival (PFS) rates of 40–50%, in contrast to approximately 75% for those with GCB-subtype tumors [1,4]. Beyond cell-of-origin classification, structural genomic aberrations further stratify clinical risk. The 2016 WHO revised classification formally recognizes a highly aggressive category—accounting for 4–8% of DLBCL cases—defined by concurrent MYC and BCL2 and/or BCL6 rearrangements detected by fluorescence in situ hybridization (FISH) and designated as high-grade B-cell lymphoma, commonly referred to as double-hit or triple-hit lymphoma [1,5,6]. In the largest multicenter retrospective analysis to date, comprising 311 patients from 23 centers, the median OS for this entity was 21.9 months with a 2-year OS rate of only 49% [5]. Separately, concurrent overexpression of MYC and BCL-2 proteins by immunohistochemistry (IHC), reported in 20–30% of DLBCL cases and termed double-expressor lymphoma (DEL), has been consistently linked to inferior outcomes irrespective of underlying gene rearrangement status [1,7]. These observations have collectively underscored the pressing need for treatment strategies that extend beyond the R-CHOP paradigm in molecularly defined high-risk subpopulations.

Primary mediastinal B-cell lymphoma (PMBL) is a biologically and clinically distinct entity originating from thymic B cells, representing approximately 6–10% of all large B-cell lymphomas [1,8]. The disease has a predilection for young adults, particularly women, and typically manifests as a bulky anterior mediastinal mass, frequently associated with pleural and pericardial effusions [8]. At the molecular level, PMBL exhibits key genomic features shared with nodular sclerosis Hodgkin lymphoma, including amplification of the REL proto-oncogene and the JAK2 locus, along with a distinctive gene expression profile that clearly separates it from conventional DLBCL [8,9,10]. Historically, the limited efficacy of standard immunochemotherapy as sole treatment for PMBL led to the routine incorporation of consolidative mediastinal radiotherapy [8]. However, given that this disease predominantly affects young patients, the long-term risks associated with mediastinal radiation—including cardiovascular toxicity and secondary malignancies—represent significant concerns [8,10,11]. In a landmark phase II study at the National Cancer Institute, Dunleavy et al. reported that DA-EPOCH-R achieved 5-year EFS and OS rates of 93% and 97%, respectively, at a median follow-up of 63 months, eliminating the need for consolidative radiotherapy in 96% of patients [8]. The addition of rituximab to DA-EPOCH significantly improved outcomes compared with DA-EPOCH alone [8,12], and these results were validated in a multicenter analysis of 156 patients demonstrating 3-year EFS of 85.9% and OS of 95.4% [9].

DA-EPOCH-R combines rituximab with infusional etoposide, doxorubicin, and vincristine alongside bolus cyclophosphamide and prednisone, with cytotoxic doses titrated cycle-by-cycle to the neutrophil nadir; this design aims to maximize tumor-cell exposure to prolonged drug concentrations while individualizing intensity to marrow tolerance. The DA-EPOCH platform was designed on the pharmacodynamic rationale that continuous 96 h infusion of etoposide, doxorubicin, and vincristine, coupled with individualized dose titration based on nadir neutrophil counts, optimizes cytotoxic exposure while maintaining a manageable toxicity profile [12]. In the original phase II trial, this strategy yielded a CR rate of 92% with PFS and OS rates of 70% and 73%, respectively [12]. In the largest multicenter retrospective analysis of 311 DHL patients, DA-EPOCH-R was associated with significantly improved PFS compared with R-CHOP (median PFS 21.6 vs. 7.8 months; *p* = 0.001) and yielded the highest CR rates among evaluated regimens [5]. However, the phase III Alliance/CALGB 50303 trial failed to demonstrate a significant PFS difference between DA-EPOCH-R and R-CHOP in unselected DLBCL (HR 0.93; 95% CI, 0.68–1.27; *p* = 0.65) [13], although high-risk molecular subgroups were markedly underrepresented and post hoc analysis suggested a benefit in patients with IPI scores of 3–5 (HR 0.63; *p* = 0.041) [13,14]. These findings have underscored the importance of identifying patient populations most likely to benefit from dose-intensive therapy.

While prospective trials have provided invaluable insights, their strict eligibility criteria and enrollment of predominantly favorable-risk populations limit the generalizability of findings to everyday clinical practice [13,15]. Real-world data reflecting diverse molecular profiles, varying comorbidity burdens, and outcomes stratified by comprehensive risk indices remain limited. To address this gap, we conducted a multicenter retrospective cohort study including 140 patients with DLBCL or PMBL treated with DA-EPOCH-R across multiple institutions in Turkey between January 2015 and December 2020. The primary objectives of this study were to evaluate the efficacy and safety of DA-EPOCH-R in a real-world setting, with particular emphasis on EFS and OS outcomes stratified by IPI risk groups and molecular subtypes, including DEL, triple-expressor, double-hit, and triple-hit lymphomas. Additionally, we sought to characterize dose-modification patterns across treatment cycles and to assess whether DA-EPOCH-R could maintain its therapeutic efficacy while reducing the need for consolidative radiotherapy, particularly in the PMBL subgroup.

## 2. Materials and Methods

### 2.1. Study Design and Population

This multicenter retrospective cohort study analyzed consecutive patients with newly diagnosed aggressive B-cell non-Hodgkin lymphoma who received dose-adjusted etoposide, prednisone, vincristine, cyclophosphamide, doxorubicin, and rituximab (DA-EPOCH-R) as first-line therapy between January 2015 and December 2020 at five academic centers in Turkey: Cukurova University Faculty of Medicine (Adana), Gülhane Training and Research Hospital (Ankara), Hacettepe University Faculty of Medicine (Ankara), Koc University Hospital (Istanbul), and Baskent University Hospital (Adana). Eligible patients were aged ≥18 years with histologically confirmed diffuse large B-cell lymphoma (DLBCL), primary mediastinal B-cell lymphoma (PMBL), Burkitt lymphoma, gray zone lymphoma, or other aggressive B-cell lymphoma subtypes according to the 2016 World Health Organization (WHO) classification of lymphoid neoplasms [6]. Patients who had received prior systemic therapy for lymphoma were excluded. Clinical data were retrospectively collected from electronic medical records and included demographic characteristics, disease-related variables, treatment details, response assessments, toxicity profiles, and survival outcomes.

The study was approved by the Institutional Review Board of Cukurova University Faculty of Medicine (approval number: decision no. 32, meeting no. 119, dated 4 February 2022). Given the retrospective nature of the study, the requirement for written informed consent was waived by the ethics committee. The study was conducted in accordance with the Declaration of Helsinki and the principles of Good Clinical Practice.

### 2.2. Treatment Protocol

DA-EPOCH-R was administered according to the standard protocol originally described by Wilson et al. [12]. Briefly, the regimen consisted of a 96 h continuous infusion of etoposide (50 mg/m^2^/day), doxorubicin (10 mg/m^2^/day), and vincristine (0.4 mg/m^2^/day, no cap) on days 1 through 4, with oral prednisone (60 mg/m^2^ twice daily) on days 1 through 5, cyclophosphamide (750 mg/m^2^) on day 5, and rituximab (375 mg/m^2^) on day 1. Cycles were repeated every 21 days for a planned total of six to eight cycles.

Dose adjustments were performed according to the pharmacodynamic paradigm described in the original protocol [12], based on the absolute neutrophil count (ANC) nadir from the preceding cycle. If the ANC nadir was above 500/µL, etoposide, doxorubicin, and cyclophosphamide doses were escalated by 20% above the previous cycle. If the ANC nadir fell below 500/µL on one or two measurements, doses were maintained at the same level as the preceding cycle. If the ANC nadir fell below 500/µL on three or more measurements, or if the platelet nadir was below 25,000/µL, doses were reduced by 20% below the preceding cycle. All patients received routine granulocyte colony-stimulating factor (G-CSF) support beginning 24 h after the completion of chemotherapy.

Consolidative radiotherapy (RT) was administered at the discretion of the treating physician, primarily guided by end-of-treatment PET/CT response and the presence of initial bulky disease. This was particularly considered in patients with PMBL who had residual metabolic activity on end-of-treatment imaging.

### 2.3. Molecular and Immunohistochemical Profiling

Immunohistochemistry (IHC) was performed on formalin-fixed paraffin-embedded (FFPE) diagnostic biopsy specimens at each participating institution as part of routine clinical practice. The following markers were assessed: MYC (c-MYC), BCL-2, BCL-6, and Ki-67 proliferation index. Positivity for each marker was determined according to the pathology reports of the respective institutions using locally established thresholds. Although the cut-offs most commonly applied for double-expressor classification are MYC ≥ 40% and BCL-2 ≥ 50% [7,16], uniform application of these thresholds across all five participating centers could not be verified retrospectively, no central pathology review or interobserver validation was performed, and digital pathology was not used. Double-expressor lymphoma (DEL) was defined as concurrent MYC and BCL-2 protein co-expression by IHC, and triple-expressor lymphoma (TEL) was defined as concurrent MYC, BCL-2, and BCL-6 co-expression, in line with established criteria [1,7].

Fluorescence in situ hybridization (FISH) analysis was performed using commercially available break-apart rearrangement probes for *MYC*, *BCL2*, and *BCL6* genes. Positivity was defined as ≥10% split signals for MYC and ≥15% for BCL2 and BCL6, in accordance with standard laboratory thresholds. Double-hit lymphoma (DHL) was defined as the presence of *MYC* rearrangement concurrent with *BCL2* and/or *BCL6* rearrangement, and triple-hit lymphoma (THL) was defined as simultaneous rearrangement of all three genes, consistent with the 2016 WHO classification of high-grade B-cell lymphoma [6].

The International Prognostic Index (IPI) was calculated for each patient at diagnosis [14] and categorized into four risk groups: low (0–1), low-intermediate (2), high-intermediate (3), and high (4–5). Ann Arbor staging was performed using contrast-enhanced computed tomography and/or 18F-fluorodeoxyglucose positron emission tomography/computed tomography (FDG-PET/CT). Bulky disease was defined as a mass ≥10 cm in its largest dimension or a mediastinal mass exceeding one-third of the transthoracic diameter.

### 2.4. Response Assessment and Endpoints

Treatment response was assessed according to the Lugano classification for response assessment of Hodgkin and non-Hodgkin lymphoma [17]. Interim response evaluation was performed after the third cycle using FDG-PET/CT. End-of-treatment response was evaluated within approximately five weeks after the completion of chemotherapy. Responses were categorized as complete response (CR), partial response (PR), stable disease (SD), or progressive disease (PD). Toxicities were graded according to the National Cancer Institute Common Terminology Criteria for Adverse Events (NCI-CTCAE) version 5.0.

The primary endpoints of this study were overall survival (OS) and event-free survival (EFS). OS was defined as the time from the first day of DA-EPOCH-R administration to death from any cause or last follow-up for censored patients. EFS was defined as the time from the first day of DA-EPOCH-R to disease progression during treatment, relapse after achieving response, death from any cause, or last follow-up, whichever occurred first. Patients who were alive without disease progression or relapse at the time of last contact were censored at that date.

### 2.5. Statistical Analysis

Descriptive statistics were used to summarize patient characteristics. Continuous variables were presented as medians with ranges, and categorical variables as frequencies with percentages. Survival estimates for OS and EFS were calculated using the Kaplan–Meier method, and differences between groups were compared using the log-rank test. The log-rank test was used for comparisons involving three or more groups. For Kaplan–Meier survival analysis by molecular subtype, patients were assigned to mutually exclusive categories in hierarchical order (THL > DHL > TEL > DEL > none) to ensure each patient contributed to a single survival curve; pairwise comparisons using overlapping molecular definitions were additionally performed where clinically indicated.

Prognostic factors for OS and EFS were evaluated using Cox proportional hazards regression analysis. Variables assessed in univariate analysis included age (≥60 vs. <60 years), sex, Ann Arbor stage (III–IV vs. I–II), IPI score (≥3 vs. <3), bulky disease, elevated lactate dehydrogenase (LDH), elevated beta-2 microglobulin (>2.5 mg/L), Ki-67 proliferation index (≥80%), serum albumin (<3.5 g/dL), diagnosis (PMBL vs. DLBCL), MYC IHC positivity, BCL-2 IHC positivity, BCL-6 IHC positivity, DEL status, TEL status, DHL status, THL status, and *MYC* FISH rearrangement status. Variables with *p* < 0.10 in univariate analysis were entered into the multivariate Cox model. Separate multivariate models were constructed for OS and EFS to account for collinearity among molecular variables, particularly the hierarchical relationship between DHL and THL and between DEL and TEL. Because all THL patients also met DHL criteria by definition, these variables could not be included simultaneously; in the OS model the high-grade genetic effect was captured by *MYC* FISH rearrangement (DHL was not entered as a separate covariate in the OS model owing to its collinearity with *MYC* FISH rearrangement and THL status), while DHL was retained in the EFS model, to avoid model instability arising from the nested relationship between these categories. The proportional hazards assumption was assessed using Schoenfeld residuals. Model discrimination was evaluated using the concordance index (C-statistic). To assess the robustness of the primary survival estimates, three sensitivity analyses were performed: (i) restricting the cohort to DLBCL and PMBL (*n* = 120); (ii) excluding patients who underwent consolidative autologous stem-cell transplantation (*n* = 25); and (iii) excluding patients who received consolidative radiotherapy (*n* = 30), the latter two to limit the influence of post-induction therapy on survival estimates.

All statistical analyses were performed using IBM SPSS Statistics version 25 (IBM Corp., Armonk, NY, USA). Figures were generated using Python version 3.12 with the matplotlib and lifelines libraries. All *p* values were two-sided, and a *p* value of <0.05 was considered statistically significant.

## 3. Results

### 3.1. Patient Characteristics

A total of 140 patients with aggressive B-cell lymphoma treated with DA-EPOCH-R at five Turkish academic centers between January 2015 and December 2020 were included in this analysis. Baseline patient and disease characteristics are summarized in Table 1. The median age at diagnosis was 44 years (range, 18–85), with a slight male predominance (57.1%). The most common histological diagnosis was DLBCL (*n* = 81; 57.9%), followed by PMBL (*n* = 39; 27.9%), Burkitt lymphoma (*n* = 5; 3.6%), gray zone lymphoma (*n* = 2; 1.4%), and other aggressive B-cell lymphoma subtypes (*n* = 13; 9.3%). The majority of patients presented with advanced-stage disease (Ann Arbor stage III–IV: 72.1%), and bulky disease was present in 45.7% of cases.

The IPI risk distribution was as follows: low (0–1) in 35 patients (25.0%), low-intermediate (2) in 42 (30.0%), high-intermediate (3) in 39 (27.9%), and high (4–5) in 24 (17.1%). On IHC, MYC protein expression was positive in 58 patients (41.4%), BCL-2 in 77 (55.0%), and BCL-6 in 80 (57.1%). FISH analysis revealed *MYC* rearrangement in 29 patients (20.7%), *BCL2* rearrangement in 23 (16.4%), and *BCL6* rearrangement in 19 (13.6%). Among molecularly defined subtypes, 39 patients (27.9%) met criteria for DEL, 21 (15.0%) for TEL, 17 (12.1%) for DHL, and 11 (7.9%) for THL.

### 3.2. Treatment Delivery and Dose Modifications

The median number of DA-EPOCH-R cycles administered was 6 (range, 1–8), with 100 patients (71.4%) completing at least six planned cycles. Dose modifications were most frequently required in cycle 2 (35% of patients receiving that cycle), with a progressive decrease in subsequent cycles: 28% in cycle 3, 22% in cycle 4, 15% in cycle 5, and 10% in cycle 6. In cycle 2, dose escalations accounted for 19 (13.8%) and dose reductions for 29 (21.0%) of modifications among patients who received the cycle, reflecting the pharmacodynamic dose-adjustment paradigm inherent to the DA-EPOCH-R regimen. The decreasing frequency of dose modifications in later cycles suggests progressive hematopoietic adaptation and is consistent with the dose-titration design of the protocol.

Following completion of DA-EPOCH-R, 59 patients (42.1%) underwent observation alone, 30 (21.4%) received consolidative radiotherapy, 25 (17.9%) proceeded to autologous stem cell transplantation (ASCT), and 26 (18.6%) received other treatments.

### 3.3. Response Assessment

Interim response evaluation after the third cycle demonstrated a CR rate of 67.9% (*n* = 95), PR in 28 patients (20.0%), SD in 6 (4.3%), and PD in 11 (7.9%). At end-of-treatment assessment, the CR rate was 68.6% (*n* = 96) and the overall response rate (ORR; CR + PR) was 82.9% (*n* = 116). SD and PD were observed in 8 (5.7%) and 16 (11.4%) patients, respectively.

CR rates varied by molecular subtype and histological diagnosis. Among patients with PMBL, the CR rate was 71.8%, compared with 63.0% in DLBCL. Molecular profiling revealed a stepwise decline in CR rates with increasing molecular complexity: DEL 56.4%, TEL 52.4%, DHL 47.1%, and THL 45.5%. Notably, even in the highest-risk THL subgroup, nearly half of the patients achieved CR with DA-EPOCH-R.

### 3.4. Survival Outcomes

#### 3.4.1. Overall Survival and Event-Free Survival

**Figure 1:**
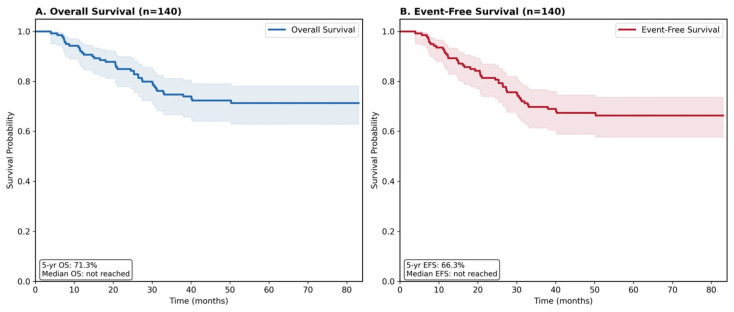
Kaplan–Meier estimates of (**A**) overall survival and (**B**) event-free survival for the entire cohort (*N* = 140) treated with dose-adjusted EPOCH-R. The median follow-up was 50.1 months. Tick marks indicate censored observations. The 5-year OS and EFS rates were 71.3% and 66.3%, respectively.

At a median follow-up of 50.1 months (range, 4–83 months), 39 patients (27.9%) had died. The median OS was not reached. The estimated 5-year OS rate for the entire cohort was 71.3%, and the 5-year EFS rate was 66.3% (Figure 1).

#### 3.4.2. Survival by IPI Risk Group

**Figure 2:**
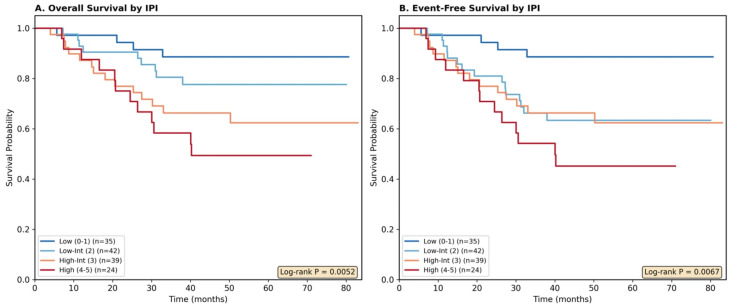
Kaplan–Meier estimates of (**A**) overall survival and (**B**) event-free survival stratified by International Prognostic Index (IPI) risk group: low (0–1; *n* = 35), low-intermediate (2; *n* = 42), high-intermediate (3; *n* = 39), and high (4–5; *n* = 24). Five-year OS rates were 88.6%, 77.6%, 62.4%, and 49.4%, respectively (log-rank *p* = 0.005). *p* values were calculated using the log-rank test.

Survival outcomes demonstrated a significant and stepwise deterioration with increasing IPI risk category. The 5-year OS rates were 88.6% for low-risk (IPI 0–1), 77.6% for low-intermediate (IPI 2), 62.4% for high-intermediate (IPI 3), and 49.4% for high-risk (IPI 4–5) patients (log-rank *p* = 0.005; Figure 2). A similar pattern was observed for EFS: 5-year EFS rates were 88.6%, 63.4%, 62.4%, and 45.1% for the four IPI risk groups, respectively (log-rank *p* = 0.007).

#### 3.4.3. Survival by Molecular Subtype

**Figure 3:**
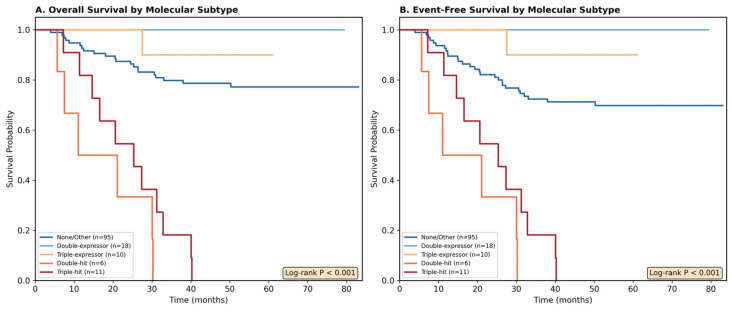
Kaplan–Meier estimates of (**A**) overall survival and (**B**) event-free survival stratified by mutually exclusive molecular subtype categories assigned in hierarchical order: none/other (*n* = 95), double-expressor lymphoma (DEL; *n* = 18), triple-expressor lymphoma (TEL; *n* = 10), double-hit lymphoma (DHL; *n* = 6), and triple-hit lymphoma (THL; *n* = 11). DHL and THL patients demonstrated 5-year OS and EFS rates approaching 0% (log-rank *p* < 0.001). *p* values were calculated using the log-rank test.

When patients were analyzed in mutually exclusive molecular categories (Figure 3), survival outcomes demonstrated a striking hierarchical pattern (log-rank *p* < 0.001). Patients in the none/other category (*n* = 95) had the most favorable outcomes, with 5-year OS and EFS rates of 77.2% and 69.8%, respectively. DEL (*n* = 18) and TEL (*n* = 10) patients had favorable outcomes comparable to or exceeding those of the none/other reference group, while DHL (*n* = 6) and THL (*n* = 11) patients demonstrated 5-year OS and EFS rates approaching 0% (log-rank *p* < 0.001 vs. the none/other reference group). Because these mutually exclusive subgroups were small (DHL, *n* = 6; THL, *n* = 11), these estimates carry wide confidence intervals and substantial statistical uncertainty and should be regarded as hypothesis-generating rather than definitive. Notably, in pairwise analysis using overlapping molecular definitions, DEL status (*n* = 39) was not associated with significantly inferior OS compared with non-DEL patients (5-year OS: 68.3% vs. 77.2%; log-rank *p* = 0.738), whereas TEL status (*n* = 21) was associated with an intermediate 5-year OS of 40.4%, reflecting the inclusion of DHL/THL patients within the overlapping TEL category.

#### 3.4.4. Survival by Histological Diagnosis: DLBCL Versus PMBL

**Figure 4:**
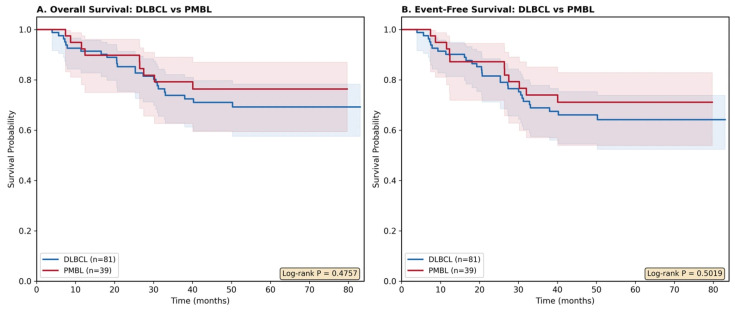
Kaplan–Meier estimates of (**A**) overall survival and (**B**) event-free survival by histological diagnosis: diffuse large B-cell lymphoma (DLBCL; *n* = 81) versus primary mediastinal B-cell lymphoma (PMBL; *n* = 39). Five-year OS rates were 69.2% and 76.4%, respectively (log-rank *p* = 0.476). *p* values were calculated using the log-rank test.

Patients with PMBL (*n* = 39) showed numerically superior survival compared with DLBCL (*n* = 81), with 5-year OS rates of 76.4% versus 69.2% and 5-year EFS rates of 71.1% versus 64.2%, although these differences did not reach statistical significance (log-rank *p* = 0.476 for OS and *p* = 0.502 for EFS; Figure 4). These results suggest comparable efficacy of DA-EPOCH-R across both histological subtypes.

#### 3.4.5. Sensitivity Analyses

In a sensitivity analysis restricted to DLBCL and PMBL (*n* = 120), 5-year OS and EFS were 71.5% and 66.4%, respectively—essentially identical to the full-cohort estimates—and DEL status remained without significant prognostic impact (5-year OS 67.4% vs. 73.3%; log-rank *p* = 0.615). In a second sensitivity analysis excluding the 25 patients who underwent consolidative ASCT (*n* = 115), 5-year OS and EFS were 69.4% and 65.1%, respectively, and the principal independent prognostic factors were unchanged: IPI ≥ 3 (HR 2.29; 95% CI, 1.13–4.64; *p* = 0.022) and MYC FISH rearrangement (HR 4.22; 95% CI, 2.14–8.30; *p* < 0.001) for OS, and DHL (HR 7.01; 95% CI, 3.59–13.71; *p* < 0.001) for EFS. In a third sensitivity analysis excluding the 30 patients who received consolidative radiotherapy (*n* = 110), 5-year OS and EFS were 69.9% and 63.5%, respectively. These analyses indicate that neither histological pooling nor post-induction radiotherapy or transplantation materially influenced the primary findings.

### 3.5. Prognostic Factor Analysis

#### 3.5.1. Univariate Cox Regression

Univariate Cox regression analysis (Table 2A) identified the following variables as significantly associated with inferior OS: IPI ≥3 (HR 2.87; 95% CI, 1.47–5.59; *p* = 0.002), BCL-6 IHC positivity (HR 2.44; 95% CI, 1.19–5.01; *p* = 0.015), TEL status (HR 2.92; 95% CI, 1.47–5.77; *p* = 0.002), THL status (HR 7.40; 95% CI, 3.62–15.09; *p* < 0.001), and *MYC* FISH rearrangement (HR 4.87; 95% CI, 2.59–9.15; *p* < 0.001). Notably, age, sex, Ann Arbor stage, bulky disease, LDH, beta-2 microglobulin, Ki-67 index, albumin, PMBL diagnosis, and DEL status were not significantly associated with OS in univariate analysis.

For EFS, significant univariate predictors included IPI ≥3 (HR 1.99; 95% CI, 1.10–3.58; *p* = 0.022), BCL-6 IHC positivity (HR 1.88; 95% CI, 1.00–3.52; *p* = 0.049), TEL status (HR 2.22; 95% CI, 1.15–4.28; *p* = 0.018), DHL (HR 7.81; 95% CI, 4.20–14.52; *p* < 0.001), THL (HR 5.59; 95% CI, 2.81–11.14; *p* < 0.001), and *MYC* FISH rearrangement (HR 3.85; 95% CI, 2.14–6.92; *p* < 0.001).

#### 3.5.2. Multivariate Cox Regression

In multivariate analysis for OS (Table 2B), IPI ≥ 3 (HR 2.54; 95% CI, 1.30–4.98; *p* = 0.007) and *MYC* FISH rearrangement (HR 3.62; 95% CI, 1.84–7.10; *p* < 0.001) emerged as independent prognostic factors, while TEL status showed a trend toward significance (HR 1.99; 95% CI, 0.97–4.12; *p* = 0.062). The concordance index for the OS model was 0.744, indicating good discriminative ability.

For EFS, multivariate analysis identified DHL as the dominant independent prognostic factor (HR 7.53; 95% CI, 2.18–25.96; *p* = 0.001), while IPI ≥ 3 showed a trend toward significance (HR 1.71; 95% CI, 0.94–3.09; *p* = 0.078). Interestingly, *MYC* FISH rearrangement lost its independent prognostic significance for EFS in the multivariate model (HR 0.96; 95% CI, 0.29–3.17; *p* = 0.941), suggesting that its effect on EFS was largely captured by DHL status. Because virtually all MYC FISH–rearranged cases in this model also met DHL criteria, these two variables were strongly collinear; the apparent loss of independent prognostic value for MYC FISH therefore reflects shared variance with DHL rather than a true absence of effect. The concordance index for the EFS model was 0.720.

### 3.6. Safety and Toxicity

Treatment-related adverse events were observed in 138 of 140 patients (98.6%) (Table 3). The most common grade 3–4 hematological toxicities were neutropenia (57.1%), thrombocytopenia (34.3%), and anemia (26.4%). Febrile neutropenia of any grade occurred in 28 patients (20.0%), with grade 3–4 febrile neutropenia in 5 patients (3.6%). Among non-hematological toxicities, grade 3–4 hepatotoxicity (AST/ALT elevation) was observed in 18 patients (12.9%), grade 3–4 mucositis in 2 (1.4%), and no grade 3–4 cardiotoxicity was reported.

Molecular subtype was associated with hematological toxicity burden. Grade 3–4 neutropenia was more frequent among DEL patients (69.2%) compared with non-DEL patients (52.5%).

## 4. Discussion

In this multicenter retrospective cohort study of 140 patients with aggressive B-cell lymphoma treated with DA-EPOCH-R at five Turkish academic centers, we demonstrate favorable long-term outcomes with 5-year OS and EFS rates of 71.3% and 66.3%, respectively, at a median follow-up of 50.1 months. The overall CR rate was 68.6%, and the ORR was 82.9%. IPI score and FISH-defined genetic rearrangements were identified as the dominant independent prognostic factors. Notably, DEL status by IHC did not confer a significant survival disadvantage in this cohort, whereas DHL and THL were associated with markedly inferior outcomes. These findings add to the growing body of real-world evidence supporting the efficacy of DA-EPOCH-R in molecularly defined high-risk B-cell lymphomas and provide the first comprehensive multicenter analysis from Turkey.

The overall survival outcomes observed in our study are consistent with those reported in landmark prospective trials and large retrospective series. In the original phase II study by Wilson et al. [12], DA-EPOCH achieved a CR rate of 92% in untreated aggressive lymphomas, establishing the pharmacodynamic rationale for the regimen. The NCI phase II study of DA-EPOCH-R in MYC-rearranged DLBCL reported a 4-year OS of 77% for the entire cohort and 82% for DHL patients [18], which led to the incorporation of DA-EPOCH-R into NCCN guidelines for high-grade B-cell lymphomas [19]. Our 5-year OS of 71.3% for a heterogeneous, unselected real-world population compares favorably with these figures, particularly given that our cohort included patients with Burkitt lymphoma, gray zone lymphoma, and other aggressive subtypes in addition to DLBCL and PMBL. Similarly, a large Asian multi-ethnic real-world analysis of 1071 DLBCL patients reported 5-year PFS and OS rates of 64.5% and 74.7%, respectively, for patients receiving rituximab-based regimens including EPOCH-R [20]. A recent analysis of over 6000 DLBCL patients in the United States demonstrated that DA-EPOCH-R was associated with improved OS among DHL/THL patients compared with R-CHOP, providing further real-world support for the use of this regimen in molecularly high-risk disease [21]. Of note, the phase III Alliance/CALGB 50303 trial did not show a survival benefit for DA-EPOCH-R over R-CHOP in an unselected DLBCL population [13], underscoring the importance of molecular stratification in treatment selection.

Perhaps the most clinically relevant finding of our study is that DEL status did not significantly impact OS (log-rank *p* = 0.738) or EFS (*p* = 0.628) in the context of DA-EPOCH-R therapy. This observation contrasts sharply with the well-established poor prognosis of DEL when treated with R-CHOP, where 5-year OS rates of 30–36% have been consistently reported [7,16]. In a meta-analysis of DEL patients, DA-EPOCH-R was found to improve PFS and OS compared with R-CHOP, with the benefit being most pronounced in younger patients [22]. Our DEL patients achieved a 5-year OS of 68.3% and a CR rate of 56.4%. This observation is consistent with—but does not establish—a benefit of dose-intensive infusional therapy in this subgroup; as cell-of-origin and rearrangement status were not fully characterized and no mechanistic correlates were measured, the present design cannot demonstrate that the regimen’s pharmacodynamic properties causally attenuate the impact of dual protein co-expression. This interpretation was further reinforced by our hierarchical analysis, in which DEL patients from whom DHL/THL cases were excluded (*n* = 18) demonstrated survival outcomes comparable to or exceeding those of the none/other reference group, suggesting that, in this cohort, protein co-expression was not associated with treatment resistance; however, this association does not by itself confirm a regimen-specific effect. Importantly, our DEL analysis could not account for cell-of-origin, a fundamental prognostic determinant in DLBCL; prior evidence suggests the benefit of DA-EPOCH-R in DEL may be largely confined to the GCB subtype [23], and the absence of these data renders our DEL findings preliminary. These results are concordant with the Italian multicenter experience reported by Dodero et al. [24], who demonstrated that DA-EPOCH-R improved outcomes in DEL patients younger than 65 years compared with R-CHOP (2-year PFS 82% vs. 43%; *p* = 0.020). The same Italian group subsequently expanded their analysis to 122 patients encompassing both DEL and DHL/THL subtypes, reporting durable 2-year outcomes (PFS 74%; OS 84%) and notably demonstrating that TP53 mutational status emerged as a key prognostic variable within this molecularly high-risk population [25]. However, some retrospective analyses have not demonstrated a clear benefit of DA-EPOCH-R over R-CHOP in DEL [26], and a multicenter University of California analysis of 155 DEL patients found similar 3-year OS rates between the two regimens [26]. Zhang et al. observed that the survival advantage conferred by DA-EPOCH-R was limited to specific clinical and molecular subgroups—namely younger age, germinal center phenotype, and higher IPI risk categories—rather than extending uniformly across the entire DEL cohort [23]. These conflicting results highlight the need for prospective randomized trials specifically designed for DEL.

In contrast to the apparent lack of an adverse DEL effect in this cohort, FISH-defined genetic aberrations retained their strong adverse prognostic significance. DHL was the dominant independent predictor of inferior EFS in multivariate analysis (HR 7.53; *p* = 0.001), and THL was associated with the highest univariate HR for OS (7.40; *p* < 0.001). Both DHL and THL subgroups demonstrated 5-year OS rates approaching 0%(albeit in very small subgroups with correspondingly wide confidence intervals), underscoring the biological aggressiveness of these entities even in the context of dose-intensive immunochemotherapy. These findings are consistent with large retrospective series in which DHL treated with R-CHOP demonstrated 5-year OS rates of only 22–27% [5,19], and meta-analyses comparing R-CHOP with more intensive regimens in DHL, including DA-EPOCH-R, have shown improved PFS but inconsistent OS benefits [5]. The observation that *MYC* FISH rearrangement was an independent prognostic factor for OS (HR 3.62; *p* < 0.001) but not for EFS in multivariate analysis—where DHL absorbed its effect—further supports the hierarchical nature of these molecular risk factors and the mechanistic distinction between protein co-expression and gene rearrangement [1,6]. Ennishi et al. [27] developed a 104-gene transcriptomic classifier (DHITsig) through gene expression profiling, revealing that approximately half of DHITsig-positive GCB-DLBCL tumors lack FISH-detectable rearrangements, thereby implying that conventional cytogenetic assessment may substantially underestimate the true burden of double-hit biology. Although BCL-6 IHC positivity was associated with inferior OS and EFS in univariate analysis, it did not retain independent significance in the multivariate models. This likely reflects its collinearity with the triple-expressor phenotype, of which BCL-6 expression is a defining component, as well as shared variance with the IPI; a confounded rather than truly independent prognostic role for BCL-6 is consistent with its variable behavior across prior DLBCL series.

The IPI retained its strong independent prognostic value in our cohort, with IPI ≥ 3 being a significant predictor of both OS (HR 2.54; *p* = 0.007) and showing a trend for EFS (HR 1.71; *p* = 0.078) in multivariate analysis. The 5-year OS rates demonstrated a clear stepwise decline from 88.6% in the low-risk group to 49.4% in the high-risk group (log-rank *p* = 0.005). This observation reaffirms the prognostic utility of the IPI, originally established over three decades ago [14], even in the era of molecularly defined risk stratification and dose-intensive immunochemotherapy. These results are consistent with recent real-world data demonstrating that IPI remains one of the most robust clinical prognostic tools across diverse DLBCL treatment settings [28].

Patients with PMBL demonstrated outcomes comparable to those with DLBCL (5-year OS: 76.4% vs. 69.2%; *p* = 0.476), with a CR rate of 71.8%. These results are consistent with the landmark NCI experience by Dunleavy et al. [8], who reported EFS and OS rates of 93% and 97%, respectively, for PMBL patients treated with DA-EPOCH-R, albeit in a highly selected prospective cohort. The multicenter validation by Giulino-Roth et al. [9] reported 3-year EFS of 85.9% and OS of 95.4% in a confirmatory analysis. The lower survival rates in our series likely reflect the unselected, real-world nature of our population, which included patients with high IPI scores and advanced-stage disease. Importantly, our data demonstrate that the favorable efficacy of DA-EPOCH-R in PMBL is maintained in a Turkish multicenter real-world setting, supporting its continued use as a preferred frontline regimen for this histological subtype and potentially reducing the need for consolidative mediastinal radiotherapy, thereby avoiding long-term radiation-associated toxicities including secondary malignancies and cardiovascular complications [10,11].

The toxicity profile observed in our study was consistent with the known safety profile of DA-EPOCH-R. Grade 3–4 neutropenia occurred in 57.1% of patients, which is comparable to rates reported in prospective trials (50–60%) [12,13]. The finding that DEL patients experienced a higher rate of grade 3–4 neutropenia (69.2% vs. 52.5% in non-DEL patients) is biologically plausible given the higher proliferative index and tumor burden associated with MYC-expressing tumors. Importantly, no grade 3–4 cardiotoxicity was observed despite the use of continuous-infusion doxorubicin, and the overall safety profile was manageable. The decreasing frequency of dose modifications from cycle 2 (35%) to cycle 6 (10%) reflects the pharmacodynamic dose-adjustment paradigm inherent to the regimen and suggests progressive hematopoietic adaptation over the course of treatment.

The treatment landscape for DLBCL is rapidly evolving. The phase III POLARIX trial has established polatuzumab vedotin plus R-CHP (pola-R-CHP) as a new frontline standard for intermediate- to high-risk DLBCL, with improved PFS compared with R-CHOP (HR 0.73; *p* = 0.02) [29]. However, POLARIX excluded PMBL patients and was not powered for molecular subgroup analyses. Whether pola-R-CHP offers advantages over DA-EPOCH-R in specific molecular subsets, particularly DHL/THL, remains an open question that warrants further investigation. Additionally, the role of chimeric antigen receptor (CAR) T-cell therapy in the second-line and beyond is increasingly recognized [30], and its potential integration into earlier treatment lines may further reshape the management of high-risk aggressive lymphomas. Our data underscore the continued relevance of molecular profiling at diagnosis to guide risk-adapted treatment strategies.

This study has several limitations that should be acknowledged. First, the inherent limitations of the retrospective study design, including potential selection bias and the inability to establish causal relationships, must be considered. Second, immunohistochemical profiling was performed locally using thresholds that could not be retrospectively harmonized, and no central pathology review was undertaken; because DEL and TEL classification depends directly on these thresholds, inter-laboratory variability may have caused misclassification in either direction, and our finding that DEL did not confer a survival disadvantage must therefore be interpreted with corresponding caution. Third, cell-of-origin classification by the Hans algorithm [31] was not systematically performed, precluding GCB versus ABC analysis—a key prognostic axis directly relevant to interpreting the DEL findings. Fourth, the sample size, while adequate for the primary analyses, limited the statistical power for subgroup analyses, particularly for the DHL (*n* = 17) and THL (*n* = 11) subgroups, as reflected in the wide confidence intervals observed in the Cox regression models. Fifth, a proportion of patients received consolidative radiotherapy or autologous stem-cell transplantation after induction; although sensitivity analyses excluding transplant or radiotherapy recipients yielded materially unchanged estimates, residual confounding by post-induction therapy cannot be fully excluded. Finally, data on CNS prophylaxis practices were not available; this is of particular relevance given the inclusion of MYC-rearranged and DHL/THL cases, which carry an elevated risk of central nervous system relapse, and the potential influence of CNS-directed management on outcomes in these subgroups therefore could not be assessed. Notwithstanding these limitations, this study represents the largest multicenter analysis of DA-EPOCH-R from Turkey, with comprehensive molecular profiling and mature follow-up of 50.1 months.

## 5. Conclusions

In conclusion, this multicenter real-world analysis demonstrates that DA-EPOCH-R provides durable long-term outcomes in patients with aggressive B-cell lymphomas, with 5-year OS and EFS rates of 71.3% and 66.3%, respectively. IPI score and FISH-defined *MYC* rearrangement were independent predictors of OS, while DHL status was the dominant prognostic factor for EFS. The observation that DEL status was not associated with a significant survival disadvantage is hypothesis-generating and may reflect the activity of dose-intensive infusional therapy, although the present design cannot establish this mechanism. These findings support the continued use of DA-EPOCH-R as a preferred regimen for molecularly high-risk aggressive B-cell lymphomas, while highlighting the need for novel therapeutic approaches—including antibody–drug conjugates and cellular therapies—for patients with FISH-defined DHL and THL who continue to experience poor outcomes even with dose-intensive immunochemotherapy, although the small number of patients in these subgroups warrants confirmation in larger series.

## Figures and Tables

**Table 1:** Baseline Patient and Disease Characteristics (*N* = 140).

Characteristic	*n* (%) or Median (Range)
**Demographics**	
Age, years, median (range)	44 (18–85)
<60 years	102 (72.9)
≥60 years	38 (27.1)
Sex: Male	80 (57.1)
Sex: Female	60 (42.9)
**Disease Characteristics**	
DLBCL	81 (57.9)
PMBL	39 (27.9)
Burkitt lymphoma	5 (3.6)
Gray zone lymphoma	2 (1.4)
Other	13 (9.3)
Stage III–IV	101 (72.1)
Bulky disease (≥10 cm)	64 (45.7)
**IPI Risk Group**	
Low (0–1)	35 (25.0)
Low-intermediate (2)	42 (30.0)
High-intermediate (3)	39 (27.9)
High (4–5)	24 (17.1)
IHC: MYC positive	58 (41.4)
IHC: BCL-2 positive	77 (55.0)
IHC: BCL-6 positive	80 (57.1)
FISH: MYC rearrangement	29 (20.7)
FISH: BCL2 rearrangement	23 (16.4)
FISH: BCL6 rearrangement	19 (13.6)
**Molecular Subtypes**	
DEL	39 (27.9)
TEL	21 (15.0)
DHL	17 (12.1)
THL	11 (7.9)
Total cycles, median (range)	6 (1–8)
Completed ≥6 cycles	100 (71.4)
CR	96 (68.6)
ORR (CR + PR)	116 (82.9)

Abbreviations: DLBCL, diffuse large B-cell lymphoma; PMBL, primary mediastinal B-cell lymphoma; IPI, International Prognostic Index; IHC, immunohistochemistry; FISH, fluorescence in situ hybridization; DEL, double-expressor lymphoma; TEL, triple-expressor lymphoma; DHL, double-hit lymphoma; THL, triple-hit lymphoma; CR, complete response; ORR, overall response rate; PR, partial response.

**Table 2:** Univariate and Multivariate Cox Proportional Hazards Regression Analysis.

**A. Univariate Analysis**					
**Variable**	***n***	**OS: HR (95% CI)**	***p***	**EFS: HR (95% CI)**	***p***
Age ≥ 60 years	38	0.75 (0.36–1.59)	0.458	0.80 (0.41–1.58)	0.530
Male sex	80	1.56 (0.80–3.04)	0.191	1.50 (0.82–2.75)	0.190
Stage III–IV	101	1.13 (0.55–2.31)	0.747	1.10 (0.57–2.13)	0.769
IPI ≥ 3	63	2.87 (1.47–5.59)	0.002	1.99 (1.10–3.58)	0.022
Bulky disease	64	0.81 (0.43–1.54)	0.520	0.90 (0.50–1.62)	0.730
Elevated LDH	93	1.11 (0.56–2.18)	0.771	1.29 (0.68–2.45)	0.439
β2-microglobulin > 2.5	54	0.97 (0.51–1.86)	0.935	1.01 (0.56–1.82)	0.982
Ki-67 ≥ 80%	63	1.34 (0.71–2.50)	0.366	1.58 (0.88–2.82)	0.122
Albumin < 3.5 g/dL	45	0.82 (0.41–1.64)	0.569	0.82 (0.43–1.56)	0.545
PMBL vs. DLBCL	39	0.75 (0.36–1.58)	0.447	0.78 (0.40–1.54)	0.477
MYC IHC (+)	58	1.53 (0.82–2.87)	0.182	1.33 (0.75–2.38)	0.331
BCL-2 IHC (+)	77	0.82 (0.44–1.53)	0.532	0.67 (0.38–1.20)	0.179
BCL-6 IHC (+)	80	2.44 (1.19–5.01)	0.015	1.88 (1.00–3.52)	0.049
DEL	39	1.12 (0.57–2.22)	0.738	0.85 (0.44–1.64)	0.628
TEL	21	2.92 (1.47–5.77)	0.002	2.22 (1.15–4.28)	0.018
THL	11	7.40 (3.62–15.09)	<0.001	5.59 (2.81–11.14)	<0.001
MYC FISH (+)	29	4.87 (2.59–9.15)	<0.001	3.85 (2.14–6.92)	<0.001
DHL	17	—	—	7.81 (4.20–14.52)	<0.001
**B. Multivariate Analysis**					
**OS Model (Concordance: 0.744)**					
**Variable**	**HR (95% CI)**		***p***		
IPI ≥ 3	2.54 (1.30–4.98)		0.007		
TEL	1.99 (0.97–4.12)		0.062		
MYC FISH (+)	3.62 (1.84–7.10)		<0.001		
**EFS Model (Concordance: 0.720)**					
**Variable**	**HR (95% CI)**		***p***		
IPI ≥ 3	1.71 (0.94–3.09)		0.078		
DHL	7.53 (2.18–25.96)		0.001		
MYC FISH (+)	0.96 (0.29–3.17)		0.941		

em dash (—) indicates that the variable was not entered/reported for that model due to collinearity or non-applicability.

**Table 3:** Treatment-Related Adverse Events by Grade and Double-Expressor Status (*N* = 140).

Adverse Event	Any Grade, *n* (%)	Grade 3–4, *n* (%)	DEL G3–4	Non-DEL G3–4
Neutropenia	91 (65.0)	80 (57.1)	27/39 (69.2)	53/101 (52.5)
Thrombocytopenia	66 (47.1)	48 (34.3)	17/39 (43.6)	31/101 (30.7)
Anemia	66 (47.1)	37 (26.4)	17/39 (43.6)	20/101 (19.8)
Febrile neutropenia	28 (20.0)	5 (3.6)	1/39 (2.6)	4/101 (4.0)
Hepatotoxicity	49 (35.0)	18 (12.9)	5/39 (12.8)	13/101 (12.9)
Nephrotoxicity	17 (12.1)	4 (2.9)	0/39 (0.0)	4/101 (4.0)
Mucositis	26 (18.6)	2 (1.4)	0/39 (0.0)	2/101 (2.0)
Cardiotoxicity	10 (7.1)	0 (0.0)	0/39 (0.0)	0/101 (0.0)

Adverse events were graded according to NCI-CTCAE version 5.0. DEL, double-expressor lymphoma. DEL group: *n* = 39; Non-DEL group: *n* = 101.

## Data Availability

The datasets generated and/or analyzed during the current study are not publicly available due to ethical reasons but are available from the corresponding author upon reasonable request.
